# Vutrisiran in Transthyretin Amyloidosis: A Pooled Safety Analysis of HELIOS-A and HELIOS-B

**DOI:** 10.1016/j.jacadv.2025.102066

**Published:** 2025-08-22

**Authors:** Ronald M. Witteles, Pablo Garcia-Pavia, Caroline Morbach, Julian D. Gillmore, Mark S. Taylor, Isabel Conceição, William B. White, Cynthia Kwok, Marianne T. Sweetser, Katherine L. Boyle, David Adams

**Affiliations:** a Division of Cardiovascular Medicine and Stanford Amyloid Center, Stanford University School of Medicine, Stanford, California, USA; b Department of Cardiology, Hospital Universitario Puerta de Hierro Majadahonda, Health Research Institute of the Puerta de Hierro Majadahonda-Segovia, Centro de Investigación Biomédica en Red Enfermedades Cardiovasculares (CIBER-CV), and Centro Nacional de Investigaciones Cardiovasculares, Madrid, Spain; c Department of Clinical Research and Epidemiology, Comprehensive Heart Failure Center, and the Department of Medicine I, University Hospital Würzburg, Würzburg, Germany; d National Amyloidosis Centre, Division of Medicine, University College London, Royal Free Hospital, London, United Kingdom; e Department of Clinical Immunology, Liverpool Hospital and South West Sydney Clinical Campus, UNSW Medicine and Health, Sydney, New South Wales, Australia; f Department of Neurosciences and Mental Health, ULS de Santa Maria, CAML, Faculty of Medicine, Centre of Studies Egas Moniz, University of Lisbon, Lisbon, Portugal; g Calhoun Cardiology Center, University of Connecticut School of Medicine, Farmington, Connecticut, USA; h Alnylam Pharmaceuticals, Cambridge, Massachusetts, USA; i Department of Neurology, CHU Bicêtre, APHP, INSERM U 1195, University Paris-Saclay, Le Kremlin Bicêtre, France

**Keywords:** cardiomyopathy, polyneuropathy, safety, transthyretin amyloidosis, vutrisiran

## Abstract

**Background:**

Vutrisiran is an RNA interference therapeutic that has demonstrated efficacy for the treatment of patients with transthyretin amyloidosis (ATTR) with polyneuropathy and cardiomyopathy in the phase 3 HELIOS-A and HELIOS-B studies, respectively. During the initial randomized treatment periods of these studies, vutrisiran was well tolerated and had an acceptable safety profile.

**Objectives:**

This pooled safety analysis aimed to evaluate the safety of vutrisiran in a large population of patients with hereditary ATTR and wild-type ATTR who received treatment for up to 58 months in HELIOS-A and HELIOS-B.

**Methods:**

Data from patients who received at least one dose of vutrisiran at any time during the HELIOS-A or HELIOS-B studies were included in this pooled safety analysis. For reference, data from the external placebo arm of HELIOS-A (APOLLO placebo) and the placebo arm of HELIOS-B studies were included. Exposure-adjusted adverse event rates (AERs) and serious AERs were calculated per 100 patient-years.

**Results:**

The combined vutrisiran group comprised 707 patients (HELIOS-A vutrisiran group: n = 160; HELIOS-B vutrisiran group: n = 547) with a mean age at symptom onset of 68.5 years; 30.3% had hereditary ATTR. These patients had a median (range) treatment duration of 33.4 months (0.0-57.8) and a cumulative exposure of 1,518.9 patient-years. In the combined vutrisiran group, AERs and serious AERs for specific adverse events were comparable to those reported in either of the placebo reference groups and those previously reported during the initial randomized treatment periods of the HELIOS-A and HELIOS-B studies. AERs for cardiac failure, the most common adverse event in vutrisiran-treated patients excluding COVID-19, were lower in the HELIOS-A and HELIOS-B vutrisiran groups than in the corresponding placebo groups. Injection-site reactions were infrequent and mild or moderate in severity. There were no ocular, hepatic, or renal safety concerns with vutrisiran treatment.

**Conclusions:**

In a broad population of patients with ATTR who were treated for up to 58 months, vutrisiran was well tolerated and had an acceptable safety profile, consistent with that previously reported for the HELIOS-A and HELIOS-B studies.

Transthyretin amyloidosis (ATTR) is a progressive, often fatal, multisystem disease caused by the deposition of toxic misfolded transthyretin (TTR) as amyloid fibrils in tissues and organs throughout the body.^1^^,^^2^ Under physiologic conditions, TTR is produced primarily in the liver and circulates as a homotetrameric protein complex that transports thyroxine and retinol,^1^^,^^3^^,^^4^ but in patients with ATTR, TTR is destabilized and aggregates into amyloid fibrils.^1^^,^^5^ ATTR is either hereditary (ATTRv), caused by inherited variants of the *TTR* gene, or occurs with aging in patients with wild-type ATTR (ATTRwt).^1^^,^^6^ Patients with ATTR can clinically present with primary cardiomyopathy (ATTR-CM) or polyneuropathy involving peripheral and autonomic nerves (ATTR-PN),^5^ but symptoms often overlap and a mixed phenotype of both cardiomyopathy and neuropathy is becoming increasingly recognized.^1^^,^^5^^,^^6^

Without disease-modifying treatment, ATTR is typically a rapidly progressive disease. Patients with hereditary transthyretin amyloidosis cardiomyopathy (ATTRv-CM) or ATTRwt have a median survival time after diagnosis of 2.6 to 4.7 years and 2.5 to 5.5 years, respectively.^7^, ^8^, ^9^, ^10^, ^11^, ^12^, ^13^, ^14^, ^15^, ^16^, ^17^, ^18^ One approach to treatment is the use of the approved TTR stabilizers tafamidis^19^^,^^20^ and acoramidis,^21^ which prevent TTR dissociation and reduce the accumulation of toxic amyloid fibrils.^1^ Diflunisal, a nonsteroidal anti-inflammatory drug, has also shown TTR-stabilizing properties and has been used off-label for patients with ATTR.^5^^,^^22^

Despite the availability of TTR stabilizers, effective and well-tolerated treatments that target the underlying pathophysiology of ATTR are needed to address the diverse symptomatology of the disease. An alternative therapeutic approach approved for patients with ATTR involves inhibiting TTR synthesis using either RNA interference therapeutics,^3^ such as patisiran^23^ and vutrisiran,^24^ or antisense oligonucleotide-based therapies, such as inotersen and eplontersen.^25^^,^^26^

Vutrisiran is an RNA interference therapeutic that targets hepatic synthesis of both variant and wild-type *TTR* mRNA, thereby preventing the buildup of toxic misfolded TTR amyloid protein throughout the body.^24^^,^^27^, ^28^, ^29^ It is comprised of a small interfering RNA conjugated to a triantennary *N*-acetylgalactosamine ligand that enhances delivery to the main site of TTR synthesis in the liver, resulting in high potency and metabolic stability that enables subcutaneous administration once every 3 months (Q3M).^27^^,^^30^^,^^31^ Vutrisiran is approved globally for the treatment of hereditary transthyretin amyloidosis polyneuropathy (ATTRv-PN) based on results from the phase 3 HELIOS-A study (NCT03759379) and approved in the United States for the treatment of wild-type transthyretin amyloidosis cardiomyopathy (ATTRwt-CM) or ATTRv-CM based on the results from the phase 3 HELIOS-B study (NCT04153149).^24^^,^^29^

ATTR, particularly ATTR-CM, is typically diagnosed in older adults who are likely to have an array of chronic comorbidities and may be receiving one or more concomitant medications.^1^ It is therefore important to establish the safety of available therapeutics in this heterogeneous patient population. During HELIOS-A and HELIOS-B, vutrisiran treatment was generally well tolerated, and most adverse events (AEs) were mild or moderate in severity and consistent with the natural history of ATTR. In the initial 18-month treatment period of HELIOS-A, AEs most commonly reported in patients included falls, pain in extremity, diarrhea, peripheral edema, urinary tract infection, arthralgia, and dizziness; all but pain in extremity and arthralgia occurred at a similar or lower incidence than in the external placebo group. During the initial 36-month double-blind treatment period of HELIOS-B, the incidence of AEs with vutrisiran was similar to or lower than that with placebo.^24^

Here, we describe pooled safety data from patients with ATTR who were treated with vutrisiran for up to 58 months during the HELIOS-A and HELIOS-B studies.

## Methods

### Study designs

This pooled safety analysis includes data from patients who received at least one dose of vutrisiran at any time during the HELIOS-A and HELIOS-B studies.^24^^,^^27^ For contextualization, data are also included from the external placebo group (APOLLO placebo) for HELIOS-A and from patients who received placebo during the randomized double-blind treatment period of HELIOS-B.

**Figure 1:**
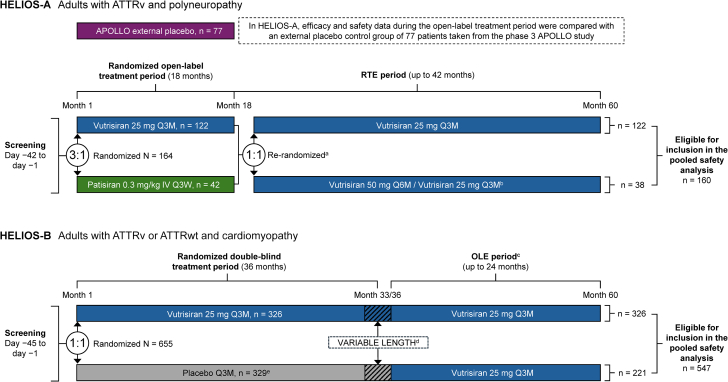
**HELIOS-A and HELIOS-B Study Designs** ^a^After the initial 18-month randomized treatment period, patients were re-randomized 1:1 to either vutrisiran 25 mg Q3M or vutrisiran 50 mg Q6M during the RTE, either directly following the randomized treatment period or at their next scheduled visit in the legacy treatment extension period. ^b^Following a protocol amendment, patients randomized to vutrisiran 50 mg Q6M were transitioned to vutrisiran 25 mg Q3M at their next study visit. ^c^During the OLE, some patients initially received vutrisiran 50 mg Q6M before being switched to 25 mg Q3M following a protocol amendment. ^d^The HELIOS-B double-blind randomized period had a variable length with a maximum duration of 33 to 36 months. ^e^One patient was randomized but not treated. ATTRv = hereditary transthyretin amyloidosis; ATTRwt = wild-type transthyretin amyloidosis; IV = intravenous; OLE = open-label extension; Q3M = once every 3 months; Q3W = once every 3 weeks; Q6M = once every 6 months; RTE = randomized treatment extension.

The study designs and primary results of HELIOS-A and HELIOS-B have been reported previously.^24^^,^^27^ Briefly, HELIOS-A was a phase 3, randomized, open-label study of vutrisiran in patients with ATTRv-PN. Enrolled patients were required to have a baseline neuropathy impairment score of 5 to 130, a polyneuropathy disability score of IIIb or lower, a Karnofsky performance status score of 60% or higher, and adequate liver and renal function. Following screening, patients were randomized 3:1 to receive vutrisiran 25 mg subcutaneously Q3M or patisiran 0.3 mg/kg intravenously once every 3 weeks, for 18 months. The primary endpoint was the change from baseline in neuropathy impairment at 9 months measured by modified Neuropathy Impairment Score +7. Data for the primary endpoint and most secondary and exploratory endpoints were compared with an external placebo control group from the APOLLO study (NCT01960348)^23^ of patisiran in ATTRv, which had similar endpoints and eligibility criteria to HELIOS-A (Figure 1). After the initial 18-month treatment period, patients from both treatment arms were re-randomized to receive vutrisiran for up to 42 months during the randomized treatment extension period (Figure 1).

HELIOS-B (NCT04153149) is a phase 3, randomized, double-blind, placebo-controlled study of vutrisiran in patients with ATTRv-CM or ATTRwt-CM.^24^ Eligible patients were required to have a diagnosis of ATTR-CM and a clinical history of heart failure. Patients who were tafamidis naïve and those receiving tafamidis at baseline were eligible to be enrolled. Initiation of on-label tafamidis was permitted in tafamidis-naïve patients during the study if considered necessary by the investigator. Following screening, patients were randomized 1:1 to receive vutrisiran 25 mg subcutaneously Q3M or placebo, for up to 36 months. The primary endpoint was a composite of all-cause mortality and recurrent cardiovascular events during the randomized double-blind treatment period. At the end of the double-blind treatment period, eligible patients could enroll in an open-label extension (OLE) period and receive vutrisiran for up to 24 months, followed by up to 12 months of follow-up. Patients who received placebo during the double-blind treatment period and continued to the OLE were switched to receive vutrisiran during the OLE (Figure 1).

The HELIOS-A randomized treatment extension period and HELIOS-B OLE period are ongoing. For the present analysis, safety data from patients treated with vutrisiran at any time during the HELIOS-A and HELIOS-B studies are included (Figure 1). For patients who initially received patisiran in the 18-month treatment period of HELIOS-A or who received placebo during the double-blind treatment period of HELIOS-B, only safety data for the period patients were receiving vutrisiran is included. As a reference to compare with the vutrisiran-treated groups, placebo data are also presented. For HELIOS-A, the external APOLLO placebo group was used. For HELIOS-B, placebo data from the double-blind treatment period are included. In HELIOS-B, some patients who received placebo during the double-blind treatment period subsequently entered the OLE and received vutrisiran. Consequently, patients included in the HELIOS-B placebo group may also be counted in the HELIOS-B vutrisiran-treated group and the combined vutrisiran-treated group for the purpose of this analysis. The maximum potential exposure to the study drug was 60 months.

For APOLLO, HELIOS-A, and HELIOS-B, the study protocol and amendments were approved by relevant Institutional Review Boards or independent ethics committees. Written informed consent was obtained from each participant. The study was conducted in accordance with all applicable regulatory requirements, the current guidelines of Good Clinical Practice, and principles originating from the Declaration of Helsinki.

## Outcomes

The primary safety outcome of HELIOS-A and HELIOS-B was the incidence of treatment-emergent AEs. For HELIOS-A and HELIOS-B, treatment-emergent AEs were defined as any AE occurring or worsening on or after the first dose of vutrisiran and through to the end of the dosing interval (ie, the earliest of 84 days after the last dose of vutrisiran or 168 days for those who received vutrisiran 50 mg once every 6 months), early study discontinuation, database cut-off, or any AE assessed as related by the investigator. A serious AE (SAE) was defined as any untoward medical occurrence that at any dose resulted in death; was life-threatening; required in-patient hospitalization or prolongation of existing hospitalization; resulted in persistent or significant disability or incapacity; resulted in a congenital anomaly or birth defect; or was a medical event that may not have resulted in death or hospitalization but may have required intervention to prevent one of the outcomes previously listed. All AEs were coded using the Medical Dictionary for Regulatory Activities (MedDRA) coding system version 23.0.

### Data and statistical analysis

The following five main groups are presented here: 1) patients who received vutrisiran at any time in HELIOS-A (hereafter the HELIOS-A vutrisiran group); 2) patients who received placebo in the APOLLO study (and whose data were subsequently used as an external reference comparator for HELIOS-A; hereafter the APOLLO placebo group); 3) patients who received vutrisiran at any time in HELIOS-B (hereafter the HELIOS-B vutrisiran group); 4) patients who received placebo in HELIOS-B (hereafter the HELIOS-B placebo group); and 5) the combined group including patients who received vutrisiran at any time in either study (hereafter the combined vutrisiran group). Where combined data are presented, patient-level data were pooled.

The cut-off dates for safety data included in the pooled analysis were September 14, 2017 for the APOLLO study (external placebo group), February 23, 2024 for HELIOS-A (vutrisiran group), and May 8, 2024 for HELIOS-B (both vutrisiran and placebo groups). For HELIOS-A, AEs with a fully or partially missing onset date were assumed to be treatment-emergent unless it could be determined unequivocally (from the partial onset date and/or a partial or complete stop date) that the event occurred before the first administration of the study drug. For HELIOS-B, AEs with a fully or partially missing onset date were imputed according to the imputation rule specified in the statistical analysis plan before determining whether they were treatment-emergent or not.

For analysis of AEs by organ system, events were grouped by MedDRA system organ class (SOC), MedDRA high-level group term, or by preferred terms identified using standardized MedDRA queries (SMQs). Cardiac events and ocular events were included for the “cardiac disorders” SOC and “eye disorders” SOC, respectively. Hepatic events included preferred terms related to the SMQ “drug-related hepatic disorders,” and renal events included preferred terms related to the SMQ “acute renal failure.”

Means and SDs are presented for continuous variables, and percentages are presented for categorical variables. AE rates (AERs) and serious AERs (SAERs) are adjusted per 100 patient-years (PY) of exposure. Data were analyzed using SAS statistical software (version 9.4 [or later], SAS Institute, Inc.).

## Results

### Study populations

A total of 707 patients were included in the combined vutrisiran group for the present pooled safety analysis (Figure 1). This comprised 160 patients with ATTR-PN in the HELIOS-A vutrisiran group and 547 patients with ATTR-CM in the HELIOS-B vutrisiran group. The HELIOS-B vutrisiran group contained 326 patients who received at least one dose of vutrisiran during the double-blind treatment period (regardless of whether they were enrolled into the OLE) and 221 patients who received at least one dose of vutrisiran in the OLE, having previously received placebo during the double-blind period. The APOLLO and HELIOS-B placebo groups included 77 and 328 patients, respectively. Patient baseline demographics and disease characteristics for each group are shown in Table 1 and Supplemental Table 1.

**Table 1:** Baseline Demographics and Disease Characteristics

	HELIOS-A		HELIOS-B		Combined Vutrisiran^b^ (N = 707)
	Vutrisiran (n = 160)	APOLLO Placebo^a^ (n = 77)	Vutrisiran^b^ (n = 547)	Placebo (n = 328)	
Age at symptom onset, y, mean (SD)	52.0 (13.6)	NR	73.3 (7.1)	73.1 (6.7)	68.5 (12.6)
Time since diagnosis, y, mean (SD)	3.9 (3.7)	2.6 (3.2)	2.6 (2.2)	1.5 (1.6)	2.9 (2.7)
Age^c^, y, mean (SD)	58.1 (12.5)	62.2 (10.8)	76.5 (6.8)	75.2 (6.3)	72.4 (11.4)
Age^c^, y, n (%)					
≥18-64	101 (63.1)	44 (57.1)	30 (5.5)	20 (6.1)	131 (18.5)
65-74	51 (31.9)	24 (31.2)	149 (27.2)	114 (34.8)	200 (28.3)
≥75	8 (5.0)	9 (11.7)	368 (67.3)	194 (59.1)	376 (53.2)
Male, n (%)	102 (63.8)	58 (75.3)	507 (92.7)	306 (93.3)	609 (86.1)
Race, n (%)					
White	113 (70.6)	50 (64.9)	465 (85.0)	275 (83.8)	578 (81.8)
Non-White^d^	47 (29.4)	26 (33.8)	71 (13.0)	45 (13.7)	118 (16.7)
Unknown	0 (0.0)	1 (1.3)	11 (2.0)	8 (2.4)	11 (1.6)
ATTR type, n (%)					
ATTRv	160 (100.0)	77 (100.0)	54 (9.9)	39 (11.9)	214 (30.3)
V30 M	73 (45.6)	40 (51.9)	4 (0.7)	2 (0.6)	77 (10.9)
V122I	6 (3.8)	1 (1.3)	35 (6.4)	25 (7.6)	41 (5.8)
ATTRwt	0 (0.0)	0 (0.0)	493 (90.1)	289 (88.1)	493 (69.7)
NYHA functional class, n (%)					
No heart failure^e^	89 (55.6)	NR	0 (0.0)	0 (0.0)	89 (12.6)
I	20 (12.5)	40 (51.9)	76 (13.9)	35 (10.7)	96 (13.6)
II	49 (30.6)	36 (46.8)	398 (72.8)	258 (78.7)	447 (63.2)
III	2 (1.3)	0 (0.0)	73 (13.3)	35 (10.7)	75 (10.6)
IV	0 (0.0)	0 (0.0)	0 (0.0)	0 (0.0)	0 (0.0)
Missing	0 (0.0)	1 (1.3)	0 (0.0)	0 (0.0)	0 (0.0)
PND score, n (%)					
0	0 (0.0)	0 (0.0)	340 (62.2)	211 (64.3)	340 (48.1)
I	56 (35.0)	20 (26.0)	154 (28.2)	91 (27.7)	210 (29.7)
II	68 (42.5)	23 (29.9)	39 (7.1)	25 (7.6)	107 (15.1)
IIIA	20 (12.5)	22 (28.6)	8 (1.5)	0 (0.0)	28 (4.0)
IIIB	16 (10.0)	11 (14.3)	6 (1.1)	0 (0.0)	22 (3.1)
IV	0 (0.0)	1 (1.3)	0 (0.0)	0 (0.0)	0 (0.0)
Missing	0 (0.0)	0 (0.0)	0 (0.0)	1 (0.3)	0 (0.0)
Baseline tafamidis use, n (%)	NA	NA	263 (48.1)	129 (39.3)	263 (37.2)
Medical history of transplant^f^, n (%)	4 (2.5)	1 (1.3)	0 (0.0)	0 (0.0)	4 (0.6)
NT-proBNP level, ng/L, median (IQR)	287.4 (66.9-945.5)	562.8 (235.5-1,580.7)^g^	2,231.0 (1,181.0-3,925.0)	1,801.0 (1,042.0-3,081.5)	1,807.0 (818.0-3,477.8)

ATTR = transthyretin amyloidosis; ATTRv = hereditary transthyretin amyloidosis; ATTRwt = wild-type transthyretin amyloidosis; NA = not applicable; NR = not reported; NT-proBNP = *N*-terminal pro B-type natriuretic peptide; NYHA = New York Heart Association; OLE = open-label extension; PND = polyneuropathy disability.

a An external placebo control arm was included using data from patients who received placebo in the phase 3 APOLLO study.

b The HELIOS-B vutrisiran group and the combined vutrisiran group contain 221 patients who originally received placebo during the HELIOS-B randomized treatment period (and are counted in the placebo group) and subsequently received vutrisiran during the OLE period.

c For the HELIOS-A vutrisiran, HELIOS-B vutrisiran, and combined vutrisiran groups, age is reported at the date patients received their first dose of vutrisiran. For the APOLLO placebo group, age is reported at the date informed consent was given. For the HELIOS-B placebo group, age is reported at the date of study randomization.

d Non-White patient populations included: Asian (N = 61), Black or African American (N = 41), Other (N = 15), More than One Race (N = 1), and Not Reported (N = 11).

e In the APOLLO study, NYHA was graded I through IV without a “no heart failure” option. In this study, NYHA functional class I included both those without heart failure and those with heart failure who did not have symptoms during ordinary physical activity.

f Included heart or renal transplant.

g n = 75.

In the combined vutrisiran group, 30.3% of patients had ATTRv and 69.7% had ATTRwt (Table 1). Among patients in the combined vutrisiran group, 11.0% had a V30 M *TTR* variant and 5.8% had a V122I *TTR* variant; this represents 36.4% and 19.2% of patients in the combined vutrisiran group with ATTRv, respectively. The proportions of patients with these *TTR* variants were similar in the placebo groups (Table 1).

Overall exposure to vutrisiran is shown for each group in Table 2. In the combined vutrisiran group, the median (range) duration of vutrisiran exposure was 33.4 (0.0-57.8) months per patient, and the cumulative exposure was 1,518.9 PY; 57.3% and 41.6% of patients were exposed to vutrisiran for at least 24 months and at least 36 months, respectively. The HELIOS-A and HELIOS-B vutrisiran groups had cumulative exposure of 539.2 PY and 979.7 PY, respectively, and the APOLLO and HELIOS-B placebo groups had cumulative exposure of 96.1 PY and 822.4 PY, respectively.

**Table 2:** Overall Exposure to Vutrisiran

Parameter	HELIOS-A		HELIOS-B		Combined Vutrisiran^b^ (N = 707)
	Vutrisiran (n = 160)	APOLLO Placebo^a^ (n = 77)	Vutrisiran^b^ (n = 547)	Placebo (n = 328)	
Duration of study drug exposure, months					
Mean (SD)	40.4 (12.9)	15.0 (5.5)	21.5 (17.2)	30.1 (9.3)	25.8 (18.2)
Median (range)	44.3 (1.7-57.8)	18.6 (1.3-18.8)	19.3 (0.0-51.6)	33.8 (1.1-37.3)	33.4 (0.0-57.8)
Cumulative exposure (patient-years)	539.2	96.1	979.7	822.4	1,518.9
Duration of study drug exposure, n (%)					
≥1 day	160 (100.0)	77 (100.0)	547 (100.0)	328 (100.0)	707 (100.0)
≥12 months	154 (96.3)	55 (71.4)	294 (53.7)	301 (91.8)	448 (63.4)
≥24 months	138 (86.3)	NA	267 (48.8)	262 (79.9)	405 (57.3)
≥36 months	111 (69.4)	NA	183 (33.5)	63 (19.2)	294 (41.6)
≥48 months	62 (38.8)	NA	4 (0.7)	NA	66 (9.3)

Abbreviations as in Table 1.

a An external placebo control arm was included using data from patients who received placebo in the phase 3 APOLLO study.

b The HELIOS-B vutrisiran group and the combined vutrisiran group contain 221 patients who originally received placebo during the HELIOS-B randomized treatment period (and are counted in the placebo group) and subsequently received vutrisiran during the OLE period.

In HELIOS-B, patients were eligible to be enrolled if they were receiving tafamidis at baseline. In the combined vutrisiran group, 37.2% of patients were receiving tafamidis at baseline (Table 1).^24^ Patients in HELIOS-A were allowed to have received prior stabilizer treatment, but were required to have stopped the treatment before the study baseline.

## Incidence of adverse events

In the combined vutrisiran group, treatment-emergent AEs were reported in 88.0% of patients: 98.8% of patients in the HELIOS-A vutrisiran group and 84.8% of patients in the HELIOS-B vutrisiran group. When adjusted for exposure, AERs in the combined vutrisiran group, the HELIOS-A vutrisiran group, and the HELIOS-B vutrisiran group were 433.8 per 100 PY, 403.6 per 100 PY, and 450.4 per 100 PY, respectively (Table 3, Central Illustration). In comparison, AERs were 1,280.5 per 100 PY for the APOLLO placebo group and 553.1 per 100 PY for the HELIOS-B placebo group (Table 3). AERs reported for patients in the HELIOS-A and HELIOS-B vutrisiran groups and the combined vutrisiran group were similar to those previously reported for patients who received vutrisiran during the initial randomized treatment periods of each study (Supplemental Table 2).^24^^,^^27^

**Table 3:** Summary of Adverse Events

	HELIOS-A			HELIOS-B			Combined	
	Vutrisiran (n = 160, 539.2 PY)		APOLLO Placebo^a^ (n = 77, 96.1 PY)	Vutrisiran (n = 547, 979.7 PY)		Placebo (n = 328, 822.4 PY)	Vutrisiran (N = 707, 1,518.9 PY)	
	n (%)	AER^b^	AER^b^	n (%)	AER^b^	AER^b^	n (%)	AER^b^
AE	158 (98.8)	403.6	1,280.5	464 (84.8)	450.4	553.1	622 (88.0)	433.8
Severe AE	62 (38.8)	27.8	91.5	199 (36.4)	47.0	62.6	261 (36.9)	40.2
SAE	77 (48.1)	38.6	103.0	254 (46.4)	66.3	76.5	331 (46.8)	56.5
AE leading to study drug discontinuation	14 (8.8)	2.6	15.6	11 (2.0)	1.3	2.2	25 (3.5)	1.8
AE leading to study withdrawal	14 (8.8)	2.6	11.4	2 (0.4)	0.2	1.1	16 (2.3)	1.1

AE = adverse event; AER = adverse event rate; PY = patient-years; SAE = serious adverse event; SAER = serious adverse event rate.

a An external placebo control arm was included using data from patients who received placebo in the phase 3 APOLLO study.

b Exposure-adjusted AER or SAER, where applicable, per 100 PY calculated as events/patient-year x 100.

**Central Illustration:**
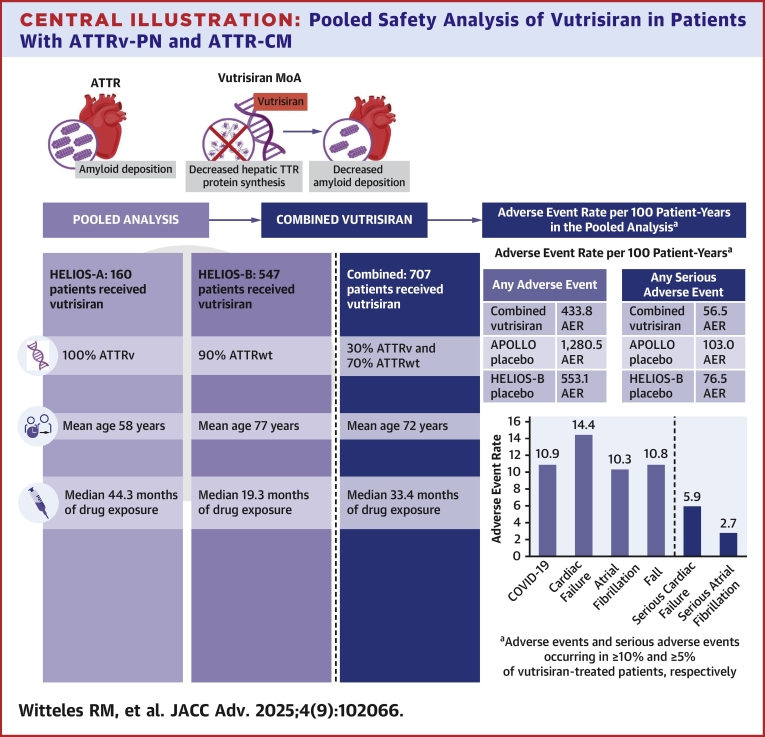
**Pooled Safety Analysis of Vutrisiran in Patients With ATTRv-PN and ATTR-CM** Pooled analysis demonstrated a consistent safety profile of vutrisiran in patients with ATTRv-PN from HELIOS-A and in patients with ATTR-CM from HELIOS-B. AER = adverse event rate; ATTR = transthyretin amyloidosis; ATTR-CM = transthyretin amyloidosis cardiomyopathy; ATTRv = hereditary transthyretin amyloidosis; ATTRv-PN = transthyretin amyloidosis polyneuropathy; ATTRwt = wild-type transthyretin amyloidosis; MoA = mechanism of action; TTR = transthyretin.

For the majority of AEs, severity was classified as mild or moderate. In the HELIOS-A vutrisiran group, the HELIOS-B vutrisiran group, and the combined vutrisiran group, severe AEs were reported in 38.8%, 36.4%, and 36.9% of patients with corresponding AERs of 27.8 per 100 PY, 47.0 per 100 PY, and 40.2 per 100 PY, respectively. Severe AERs were 91.5 per 100 PY in the APOLLO placebo group and 62.6 per 100 PY in the HELIOS-B placebo group (Table 3). In patients treated with vutrisiran, AEs leading to study drug discontinuation or study withdrawal were reported in 3.5% of patients (AER: 1.8 per 100 PY) and 2.3% of patients (AER: 1.1 per 100 PY), respectively, in the combined vutrisiran group. The most common AE leading to study drug discontinuation in the combined vutrisiran group was cardiac failure (0.8%) (Table 4). In comparison, AERs leading to study drug discontinuation or study withdrawal were 15.6 and 11.4 per 100 PY in the APOLLO placebo group and 2.2 and 1.1 per 100 PY in the HELIOS-B placebo group (Table 3).

**Table 4:** Most Common Adverse Events, Serious Adverse Events, and Adverse Events Leading to Study Drug Discontinuation

Type of AE	HELIOS-A			HELIOS-B			Combined	
	Vutrisiran (n = 160, 539.2 PY)		APOLLO Placebo^a^ (n = 77, 96.1 PY)	Vutrisiran (n = 547, 979.7 PY)		Placebo (n = 328, 822.4 PY)	Vutrisiran (N = 707, 1,518.9 PY)	
	n (%)	AER^b^	AER^b^	n (%)	AER^b^	AER^b^	n (%)	AER^b^
Individual AEs occurring in ≥10% of vutrisiran-treated patients^c^								
COVID-19	47 (29.4)	10.0	0.0	102 (18.6)	11.3	13.3	149 (21.1)	10.9
Cardiac failure	8 (5.0)	1.5	4.2	132 (24.1)	21.5	31.0	140 (19.8)	14.4
Atrial fibrillation	17 (10.6)	4.6	7.3	87 (15.9)	13.4	11.2	104 (14.7)	10.3
Fall	36 (22.5)	14.3	44.7	61 (11.2)	8.9	13.5	97 (13.7)	10.8
Serious AEs occurring in ≥5% of vutrisiran-treated patients^c^								
Cardiac failure	4 (2.5)	0.7	2.1	51 (9.3)	8.7	11.4	55 (7.8)	5.9
Atrial fibrillation	3 (1.9)	0.7	1.0	32 (5.9)	3.8	3.3	35 (5.0)	2.7
AEs leading to study drug discontinuation occurring in ≥0.5% of vutrisiran-treated patients^c^								
Cardiac failure	1 (0.6)	0.2	1.0	5 (0.9)	0.5	0.1	6 (0.8)	0.4

Preferred terms are presented by frequency in the combined vutrisiran group in descending order.

Abbreviations as in Table 3.

a An external placebo control arm was included using data from patients who received placebo in the phase 3 APOLLO study. The APOLLO study was completed before the onset of the COVID-19 pandemic.

b Exposure-adjusted AER or SAER, where applicable, per 100 PY calculated as events/patient-year x 100.

c In the combined vutrisiran-treated group.

The most common AEs and SAEs are shown in Table 4. In the combined vutrisiran group, the most common AEs were COVID-19 (21.1%; AER: 10.9 per 100 PY), cardiac failure (19.8%; AER: 14.4 per 100 PY), and atrial fibrillation (14.7%; AER: 10.3 per 100 PY). In the HELIOS-A and HELIOS-B vutrisiran groups, cardiac failure AERs were 1.5 per 100 PY and 21.5 per 100 PY, respectively. For reference, the cardiac failure AER was 4.2 per 100 PY in the APOLLO placebo group and 31.0 per 100 PY in the HELIOS-B placebo group (Table 4). Overall, the type and rates of AEs were similar to those previously reported for the initial randomized treatment periods of each study (Supplemental Table 3).

SAEs were reported in 46.8% of patients (SAER: 56.5 per 100 PY) in the combined vutrisiran group (Table 3); 33.8% of patients had at least one SAE that was classified as severe. In the HELIOS-A vutrisiran group, the HELIOS-B vutrisiran group, and the combined vutrisiran group, treatment-emergent SAEs with a fatal outcome were reported in 9.4%, 10.8%, and 10.5% of patients, respectively. The most common SAEs in the combined vutrisiran group were cardiac failure (7.8%; SAER: 5.9 per 100 PY) and atrial fibrillation (5.0%; SAER: 2.7 per 100 PY) (Table 4). Cardiac failure SAERs were 0.7 and 8.7 per 100 PY in the HELIOS-A vutrisiran group and HELIOS-B vutrisiran group, respectively; in the APOLLO and HELIOS-B placebo groups, SAERs were 2.1 and 11.4 per 100 PY, respectively (Table 4). Compared with rates previously reported in the initial randomized treatment periods of each study, SAERs were similar in patients who received vutrisiran for up to 58 months (Supplemental Table 3).

Treatment-related injection-site reactions (ISRs) and associated signs and symptoms were evaluated. In the combined vutrisiran group, 2.1% of patients (AER: 1.0 per 100 PY) experienced at least one ISR. In the HELIOS-B placebo group, 2.4% of patients (AER: 1.1 per 100 PY) experienced an ISR. The most common ISR symptom was pain at the injection site, which occurred in 1.1% of patients in the combined group. None of the reported ISRs were serious or severe, 94.7% were mild, and 5.3% were moderate in severity, and all were transient with none requiring a change in study drug administration.

In the combined vutrisiran group, 64.9% of patients had baseline and postbaseline antidrug antibody (ADA) results. Of these, 1.5% of patients developed treatment-emergent ADAs. These were low titer and transient, and overall, there was no pattern of AEs in patients with ADAs to suggest an impact of ADAs on the safety profile of vutrisiran.

Generally, the incidence of any AEs, SAEs, or cardiac failure listed as an AE did not show important differences in patient subgroups based on age (<75 or ≥75 years), sex (male or female), race (White or non-White), or ATTR type (ATTRv or ATTRwt) in the combined analysis, other than expected variations due to the natural history of the disease in certain subgroups, such as older patients (Supplemental Table 4).

## Adverse events by organ system

Cardiac AEs were evaluated in this analysis (Table 5, Supplemental Table 5). In the combined vutrisiran group, cardiac AEs were reported in 48.2% of patients (AER: 50.7 per 100 PY). The cardiac AER was 23.6 per 100 PY in the HELIOS-A vutrisiran group and 46.8 per 100 PY in the APOLLO placebo group. In the HELIOS-B vutrisiran group, the cardiac AER was 65.6 per 100 PY vs 83.5 per 100 PY in the HELIOS-B placebo group. Cardiac AEs by SOC considered serious were reported in 23.6% of patients (SAER: 18.2 per 100 PY) in the combined vutrisiran group (Supplemental Table 5) and the most common cardiac SAEs were atrial fibrillation, cardiac failure, and ventricular tachycardia. No cardiac SAEs were assessed as being related to vutrisiran by the study investigator. Thromboembolic complications were rare, with AER rates of pulmonary embolism and deep vein thrombosis of 0.3 per 100 PY each reported in the combined vutrisiran group (Supplemental Table 6).

**Table 5:** Adverse Events by Organ System

Organ System	HELIOS-A			HELIOS-B			Combined	
	Vutrisiran (n = 160, 539.2 PY)		APOLLO Placebo^a^ (n = 77, 96.1 PY)	Vutrisiran (n = 547, 979.7 PY)		Placebo (n = 328, 822.4 PY)	Vutrisiran (N = 707, 1,518.9 PY)	
	n (%)	AER^b^	AER^b^	n (%)	AER^b^	AER^b^	n (%)	AER^b^
Cardiac events^c^	58 (36.3)	23.6	46.8	283 (51.7)	65.6	83.5	341 (48.2)	50.7
Ocular events^d^	49 (30.6)	17.4	27.0	57 (10.4)	8.0	12.6	106 (15.0)	11.3
Hepatic events^e^	20 (12.5)	6.3	7.3	69 (12.6)	10.8	10.9	89 (12.6)	9.2
Renal events^f^	11 (6.9)	2.8	13.5	66 (12.1)	8.2	9.7	77 (10.9)	6.3

If a patient had more than one event in a given SOC or SMQ, that patient is counted once for the SOC or SMQ.

MedDRA = Medical Dictionary for Regulatory Activities; SMQ = standardized MedDRA query; SOC = system organ class; other abbreviations as in Table 3.

a An external placebo control arm was included using data from patients who received placebo in the phase 3 APOLLO study.

b Exposure-adjusted AER per 100 PY calculated as events/patient-year x 100.

c Cardiac disorders SOC.

d Eye disorders SOC.

e Mapped to the MedDRA “drug-related hepatic disorders” comprehensive search SMQ (broad and narrow terms).

f Mapped to the MedDRA “acute renal failure” comprehensive search SMQ (broad and narrow terms).

Ocular AEs by SOC were also evaluated in this analysis (Table 5). In the combined vutrisiran group, ocular AEs were reported in 15.0% of patients (AER: 11.3 per 100 PY). Ocular AERs were 17.4 per 100 PY and 27.0 per 100 PY in the HELIOS-A vutrisiran and APOLLO placebo groups, respectively, and 8.0 per 100 PY and 12.6 per 100 PY in the HELIOS-B vutrisiran and HELIOS-B placebo groups, respectively. The most common ocular events occurring in >2% of vutrisiran-treated patients were cataract (2.7%) and dry eye (2.1%).

Furthermore, hepatic AEs defined by the “drug-related hepatic disorders” SMQ, were evaluated (Table 5). In the combined vutrisiran group, hepatic AEs were reported in 12.6% of patients (AER: 9.2 per 100 PY). Hepatic AERs were 6.3 per 100 PY in the HELIOS-A vutrisiran group and 7.3 per 100 PY in the APOLLO placebo group. The hepatic AER in the HELIOS-B vutrisiran group was 10.8 per 100 PY vs 10.9 per 100 PY in the HELIOS-B placebo group.

Finally, renal AEs defined by the “acute renal failure” SMQ were evaluated in this analysis (Table 5). In the combined vutrisiran group, renal AEs were reported in 10.9% of patients (AER: 6.3 per 100 PY). Renal AERs were 2.8 per 100 PY in the HELIOS-A vutrisiran group and 13.5 per 100 PY in the APOLLO placebo group. The renal AER was 8.2 per 100 PY in the HELIOS-B vutrisiran group and 9.7 per 100 PY in the HELIOS-B placebo group. In addition, there were no safety concerns regarding liver function tests, hematology, or renal function related to vutrisiran based on laboratory assessments.

AERs for the organ systems reported for patients who received vutrisiran for up to 58 months in this pooled analysis were consistent with those reported for patients who received vutrisiran during the randomized periods of HELIOS-A and HELIOS-B (Supplemental Table 7).

## Discussion

In the phase 3 HELIOS-A and HELIOS-B studies, the efficacy and safety of vutrisiran were evaluated across a broad population representative of the overall global population of patients with ATTR.^24^^,^^27^ By combining data from both studies in this pooled analysis, we demonstrate that in a large group of patients who have extensive exposure to the therapy, vutrisiran is well tolerated and has an acceptable safety profile.

Although patients typically receive a diagnosis of either ATTR-PN or ATTR-CM, they can have a mixed phenotype with symptoms of both polyneuropathy and cardiomyopathy.^1^^,^^6^ The present pooled safety data included patients with a diagnosis of ATTR-PN or ATTR-CM who were enrolled in the HELIOS-A or HELIOS-B studies, respectively. However, patients with ATTR-PN often also had cardiomyopathy, and patients with ATTRv-CM often had evidence of polyneuropathy. Consequently, in this analysis, patients had a wide range of severity in neuropathy, including those with a polyneuropathy disability score of III or more, and differing degrees of cardiac involvement ranging from mild to severe, including those with NHYA class III.

This analysis also included patients with both ATTRv and ATTRwt, encompassing the spectrum of disease etiologies. Overall, in the combined vutrisiran group, approximately 30% of patients had ATTRv. These included patients with the V122I *TTR* variant, which is the most common variant observed in patients with ATTRv-CM in the United States^5^ and is associated with greater functional impairment and poorer outcomes.^11^

Furthermore, although the age of onset varies between patients with ATTR-PN and those with ATTR-CM, and between those with ATTRv and ATTRwt,^5^ ATTR is most often diagnosed in older adults.^1^ The patients evaluated in the present analysis had a broad age range, with age at symptom onset among patients who received vutrisiran of 52.0 years in those with ATTR-PN (in HELIOS-A) and 73.3 years in those with ATTR-CM (in HELIOS-B); over 80% of patients included in the combined vutrisiran group were aged 65 years or older. Among this combined vutrisiran group, 48.1% of patients in HELIOS-B were also receiving the TTR stabilizer tafamidis at baseline and continued to receive it during the study. The totality of data reported in this analysis reinforces the safety of vutrisiran in this broad population of patients who typically have chronic comorbidities and are likely to be receiving multiple concomitant medications.

During the initial randomized treatment periods of HELIOS-A and HELIOS-B, vutrisiran demonstrated an acceptable safety profile for up to 18 and 36 months of treatment, respectively;^24^^,^^27^ the majority of AEs were mild or moderate in severity and consistent with the natural history of ATTR, with the incidence of AEs being largely similar or lower in patients who received vutrisiran than in those who received placebo. In this pooled safety analysis that included over 700 patients who were treated for up to 58 months with a combined vutrisiran exposure of 1,518.9 PY, no mechanistically or clinically relevant safety concerns emerged. The types and rates of AEs observed were consistent with underlying disease pathology and similar to those observed during the initial randomized treatment periods of each study.^24^^,^^27^ Furthermore, among those who received vutrisiran in HELIOS-A and HELIOS-B, rates of treatment discontinuation or withdrawal from the study due to AEs were lower than in the corresponding placebo groups and <2% in the combined vutrisiran group. The absence of new, clinically meaningful safety events with vutrisiran reinforces earlier findings from the initial randomized treatment periods of HELIOS-A and HELIOS-B across patients with diverse ATTR phenotypes.

With respect to specific organ systems, there were no cardiac, ocular, hepatic, renal, hematologic, or immunogenicity-related safety concerns identified with vutrisiran treatment. Cardiac AERs reflected the underlying baseline disease as shown by the event rates in the combined vutrisiran group, separate HELIOS-A and HELIOS-B vutrisiran groups, and the respective placebo comparator groups.^24^^,^^27^ In addition, ocular AEs were only reported in a small number of vutrisiran-treated patients and balanced between groups. Finally, consistent with data previously reported, ISRs were largely mild and infrequent, with no increase in severity over time, and incidence of ADAs was low with vutrisiran treatment for up to 58 months.

### Study limitations

A limitation of this study is the absence of placebo reference data for the full 60-month duration of the analysis for direct comparison of AE rates. The maximum duration of placebo data was limited to the initial randomized treatment periods of APOLLO and HELIOS-B, therefore between-group comparisons are based on exposure-adjusted event rates. In addition, in HELIOS-B, patients who received placebo during the double-blind treatment period could enter the OLE and receive vutrisiran. Therefore, some patients included in the HELIOS-B placebo group were also counted in the HELIOS-B vutrisiran and the combined vutrisiran groups for the purpose of this analysis. Because safety data were only included for the period patients were receiving vutrisiran, AERs in the HELIOS-B vutrisiran group and therefore the combined vutrisiran group may have been influenced in part by patients who had previously received placebo. Nevertheless, over half of vutrisiran-treated patients in this analysis received treatment for at least 24 months.

## Conclusions

This pooled analysis, in a large and heterogeneous population of patients with ATTR who had up to 58 months of treatment, demonstrates that vutrisiran is well tolerated and has an acceptable safety profile. The nature and incidence of AEs were consistent with those previously reported for the initial randomized treatment periods of HELIOS-A and HELIOS-B.^24^^,^^27^

Perspectives **COMPETENCY IN PATIENT CARE:** In this pooled safety analysis, vutrisiran had an acceptable safety profile and was well tolerated in a broad population of patients with ATTRv and ATTRwt who received treatment for up to 58 months.

**TRANSLATIONAL OUTLOOK:** Continued monitoring of safety events will provide further long-term data on the safety of vutrisiran in patients with ATTR in clinical practice.

## Funding support and author disclosures

The study was funded by 10.13039/100006400Alnylam Pharmaceuticals. Ronald M. Witteles has received consulting fees from Alexion, Alnylam Pharmaceuticals, AstraZeneca, BridgeBio, Novo Nordisk, and Pfizer. Dr Garcia-Pavia has received speakers fees from Alnylam Pharmaceuticals, AstraZeneca, Bayer, BridgeBio, Intellia Therapeutics, Ionis Pharmaceuticals, Novo Nordisk, and Pfizer; consulting fees from Alexion, Alnylam Pharmaceuticals, AstraZeneca, ATTRalus, Bayer, BridgeBio, Intellia Therapeutics, Ionis Pharmaceuticals, Neuroimmune, Novo Nordisk, and Pfizer; and research/educational support to their institution from Alnylam Pharmaceuticals, AstraZeneca, BridgeBio, Intellia Therapeutics, Novo Nordisk, and Pfizer. Dr Morbach has received advisory fees, speakers fees, and travel grants from Alexion, Alnylam Pharmaceuticals, AstraZeneca, Bayer, Boehringer Ingelheim, EBR Systems, Edwards, Intellia Therapeutics, Janssen, Lilly, Novo Nordisk, Pfizer, SOBI, and Tomtec Imaging Systems; and serves as principal investigator in trials sponsored by Alnylam Pharmaceuticals, AstraZeneca, Bayer, Intellia Therapeutics, and Novo Nordisk. Dr Gillmore has provided advisory services for Alexion, Alnylam Pharmaceuticals, AstraZeneca, ATTRalus, BridgeBio, Intellia Therapeutics, Ionis, and Lycia; and has received an institutional grant from Alnylam Pharmaceuticals. Dr Conceição has received speaker fees from Alnylam Pharmaceuticals, AstraZeneca, Ionis Pharmaceuticals, and Pfizer; consulting fees from Alnylam Pharmaceuticals, AstraZeneca, BridgeBio, Intellia Therapeutics, Ionis Pharmaceuticals, and Pfizer; and research/educational support for their institution from Alnylam Pharmaceuticals, AstraZeneca, and Pfizer. Dr White has consulted on cardiovascular safety and been a member of the safety review committee and data safety monitoring board for Alnylam Pharmaceuticals. Drs Kwok, Sweetser, and Boyle are employees of, and hold stock or stock options in, Alnylam Pharmaceuticals. Dr Adams has received consulting fees from Alnylam Pharmaceuticals, AstraZeneca, and Intellia Therapeutics. All other authors have reported that they have no relationships relevant to the contents of this paper to disclose.

## References

[1] Hawkins P.N., Ando Y., Dispenzeri A., Gonzalez-Duarte A., Adams D., Suhr O.B. (2015). Evolving landscape in the management of transthyretin amyloidosis. Ann Med.

[2] Ando Y., Coelho T., Berk J.L. (2013). Guideline of transthyretin-related hereditary amyloidosis for clinicians. Orphanet J Rare Dis.

[3] Friedrich M., Aigner A. (2022). Therapeutic siRNA: state-of-the-art and future perspectives. BioDrugs.

[4] Liz M.A., Coelho T., Bellotti V., Fernandez-Arias M.I., Mallaina P., Obici L. (2020). A narrative review of the role of transthyretin in health and disease. Neurol Ther.

[5] Ruberg F.L., Grogan M., Hanna M., Kelly J.W., Maurer M.S. (2019). Transthyretin amyloid cardiomyopathy: JACC state-of-the-art review. J Am Coll Cardiol.

[6] Maurer M.S., Hanna M., Grogan M. (2016). Genotype and phenotype of transthyretin cardiac amyloidosis: THAOS (transthyretin amyloid outcome survey). J Am Coll Cardiol.

[7] Swiecicki P.L., Zhen D.B., Mauermann M.L. (2015). Hereditary ATTR amyloidosis: a single-institution experience with 266 patients. Amyloid.

[8] Sattianayagam P.T., Hahn A.F., Whelan C.J. (2012). Cardiac phenotype and clinical outcome of familial amyloid polyneuropathy associated with transthyretin alanine 60 variant. Eur Heart J.

[9] Gertz M.A., Kyle R.A., Thibodeau S.N. (1992). Familial amyloidosis: a study of 52 North American-born patients examined during a 30-year period. Mayo Clin Proc.

[10] Castaño A., Drachman B.M., Judge D., Maurer M.S. (2015). Natural history and therapy of TTR-cardiac amyloidosis: emerging disease-modifying therapies from organ transplantation to stabilizer and silencer drugs. Heart Fail Rev.

[11] Lane T., Fontana M., Martinez-Naharro A. (2019). Natural history, quality of life, and outcome in cardiac transthyretin amyloidosis. Circulation.

[12] Grogan M., Scott C., Kyle R.A. (2016).

[13] Siepen F.A.D., Bauer R., Voss A. (2018). Predictors of survival stratification in patients with wild-type cardiac amyloidosis. Clin Res Cardiol.

[14] Connors L.H., Sam F., Skinner M. (2016). Heart failure resulting from age-related cardiac amyloid disease associated with wild-type transthyretin: a prospective, observational cohort study. Circulation.

[15] Givens R.C., Russo C., Green P., Maurer M.S. (2013). Comparison of cardiac amyloidosis due to wild-type and V122I transthyretin in older adults referred to an academic medical center. Aging Health.

[16] Pinney J.H., Whelan C.J., Petrie A. (2013). Senile systemic amyloidosis: clinical features at presentation and outcome. J Am Heart Assoc.

[17] Ruberg F.L., Maurer M.S., Judge D.P. (2012). Prospective evaluation of the morbidity and mortality of wild-type and V122I mutant transthyretin amyloid cardiomyopathy: the Transthyretin Amyloidosis Cardiac Study (TRACS). Am Heart J.

[18] Gillmore J.D., Damy T., Fontana M. (2018). A new staging system for cardiac transthyretin amyloidosis. Eur Heart J.

[19] European Medicines Agency, Pfizer Summary of product characteristics. https://www.ema.europa.eu/en/medicines/human/EPAR/vyndaqel.

[20] U.S. Food and Drug Administration, Pfizer VYNDAMAX Va. Full prescribing information. https://www.fda.gov/media/126283/download.

[21] U.S. Food and Drug Administration, BridgeBio Pharma Inc ATTRUBY. Prescribing information. https://www.accessdata.fda.gov/drugsatfda_docs/label/2024/216540s000lbl.pdf.

[22] Berk J.L., Suhr O.B., Obici L. (2013). Repurposing diflunisal for familial amyloid polyneuropathy: a randomized clinical trial. JAMA.

[23] Adams D., Gonzalez-Duarte A., O'Riordan W.D. (2018). Patisiran, an RNAi therapeutic, for hereditary transthyretin amyloidosis. N Engl J Med.

[24] Fontana M., Berk J.L., Gillmore J.D. (2024). Vutrisiran in patients with transthyretin amyloidosis with cardiomyopathy. N Engl J Med.

[25] Benson M.D., Dasgupta N.R., Monia B.P. (2019). Inotersen (transthyretin-specific antisense oligonucleotide) for treatment of transthyretin amyloidosis. Neurodegener Dis Manag.

[26] Coelho T., Marques W., Dasgupta N.R. (2023). Eplontersen for hereditary transthyretin amyloidosis with polyneuropathy. JAMA.

[27] Adams D., Tournev I.L., Taylor M.S. (2023). Efficacy and safety of vutrisiran for patients with hereditary transthyretin-mediated amyloidosis with polyneuropathy: a randomized clinical trial. Amyloid.

[28] Habtemariam B.A., Karsten V., Attarwala H. (2021). Single-dose pharmacokinetics and pharmacodynamics of transthyretin targeting N-acetylgalactosamine-small interfering ribonucleic acid conjugate, vutrisiran, in healthy subjects. Clin Pharmacol Ther.

[29] Alnylam Pharmaceuticals Inc US prescribing information: AMVUTTRA (vutrisiran) injection, for subcutaneous use. https://www.alnylam.com/sites/default/files/pdfs/amvuttra-us-prescribing-information.pdf.

[30] European Medicines Agency, Alnylam Pharmaceuticals Inc Summary of product characteristics. https://www.ema.europa.eu/en/documents/product-information/amvuttra-epar-product-information_en.pdf.

[31] Planté-Bordeneuve V., Perrain V. (2024). Vutrisiran: a new drug in the treatment landscape of hereditary transthyretin amyloid polyneuropathy. Expert Opin Drug Discov.

