# Supplementary material for: Vutrisiran in Transthyretin Amyloidosis: A Pooled Safety Analysis of HELIOS-A and HELIOS-B
Source: JACC Adv. 2025 Aug 22;4(9):102066. doi: 10.1016/j.jacadv.2025.102066 (PMC12397930; doi:10.1016/j.jacadv.2025.102066)
Supplement: Supplemental Material [file mmc1.pdf]

SUPPLEMENTAL TABLE 1 **Baseline Echocardiography Parameters and Cardiac History During the Initial Randomized Treatment Periods**

	HELIOS-A		HELIOS-B	
	**Vutrisiran n = 122**	**APOLLO Placebo[a] n = 77**	**Vutrisiran n = 326**	**Placebo n = 328**
**Echocardiography parameters, mean (SD)**				
Mean LV wall thickness, cm	1.4 (0.4) (n = 117)	1.6 (0.3) (n = 74)	1.8 (0.3) (n = 321)	1.8 (0.3) (n = 324)
LV mass, g	209.9 (91.8) (n = 116)	248.3 (78.5) (n = 73)	351.8 (87.7) (n = 320)	350.6 (93.2) (n = 318)
Global longitudinal strain (HELIOS-A)/Average peak longitudinal strain (HELIOS-B), %	–15.8 (4.0) (n = 116)	–16.3 (3.7) (n = 72)	–14.0 (3.5) (n = 324)	–14.0 (3.5) (n = 328)
Cardiac output, L/min	3.9 (1.1) (n = 115)	4.2 (1.3) (n = 73)	NR	NR
**Cardiac history, n (%)[b]**				

Myocardial disorders	64 (52.5)	36 (46.8)	15 (4.6)	15 (4.6)
Heart failure	38 (31.1)	12 (15.6)	311 (95.4)	312 (95.1)
Cardiac conduction disorders	16 (13.1)	21 (27.3)	120 (36.8)	128 (39.0)
Supraventricular arrhythmias	12 (9.8)	14 (18.2)	222 (68.1)	224 (68.3)

[a]An external placebo control arm was included using data from patients who received placebo in the phase 3 APOLLO study.

[b]iHighHigh-level term is presented by the frequency in the HELIOS-A vutrisiran group in descending order.

LV = left ventricular; SD = standard deviation.

SUPPLEMENTAL TABLE 2 Summary of Adverse Events During the Initial Randomized Treatment Periods

	HELIOS-A 18-month Treatment Period				HELIOS-B Double-blind Treatment Period			
	Vutrisiran n = 122, 191.3 PY		APOLLO Placebo[a] n = 77, 96.1 PY		Vutrisiran n = 326, 833.9 PY		Placebo n = 328, 822.4 PY	
**Parameter**	**n (%)**	**AER[b]**	**n (%)**	**AER[b]**	**n (%)**	**AER[b]**	**n (%)**	**AER[b]**
AE	119 (97.5)	552.6	75 (97.4)	1280.5	322 (98.8)	424.7	323 (98.5)	553.1
Severe AE	19 (15.6)	20.9	28 (36.4)	91.5	158 (48.5)	43.9	194 (59.1)	62.6
SAE	32 (26.2)	32.9	31 (40.3)	103.0	201 (61.7)	63.3	220 (67.1)	76.5
AE leading to study drug discontinuation	3 (2.5)	1.6	11 (14.3)	15.6	10 (3.1)	1.2	13 (4.0)	2.2
AE leading to study withdrawal	3 (2.5)	1.6	9 (11.7)	11.4	2 (0.6)	0.2	6 (1.8)	1.1

[a]An external placebo control arm was included using data from patients who received placebo in the phase 3 APOLLO study. [b]Exposure-adjusted AER or SAER, where applicable, per 100 patient-years calculated as events/patient-year x 100.

AE = adverse event; AER = adverse event rate; PY = patient-years; SAE = serious adverse event; SAER = serious adverse event rate.

SUPPLEMENTAL TABLE 3 Adverse Events and Serious Adverse Events During the Initial Randomized Treatment Periods Matched to the Most Common Events Reported in the Pooled Analysis

	HELIOS-A 18-month Treatment Period				HELIOS-B Double-blind Treatment Period			
	Vutrisiran n = 122, 191.3 PY		APOLLO Placebo[a] n = 77, 96.1 PY		Vutrisiran n = 326, 833.9 PY		Placebo n = 328, 822.4 PY	
	n (%)	AER[b]	n (%)	AER[b]	n (%)	AER[b]	n (%)	AER[b]
**Any AEs occurring in ≥10% of vutrisiran-treated patients[c]**								
COVID-19	4 (3.3)	2.6	0 (0.0)	0.0	87 (26.7)	11.2	99 (30.2)	13.3
Cardiac failure	3 (2.5)	1.6	4 (5.2)	4.2	101 (31.0)	20.7	128 (39.0)	31.0
Atrial fibrillation	8 (6.6)	6.3	5 (6.5)	7.3	69 (21.2)	12.8	68 (20.7)	11.2
Fall	22 (18.0)	20.4	22 (28.6)	44.7	42 (12.9)	7.7	69 (21.0)	13.5
**Serious AEs occurring in ≥5% of vutrisiran-treated patients[c]**								
Cardiac failure	1 (0.8)	0.5	2 (2.6)	2.1	38 (11.7)	8.4	57 (17.4)	11.4
Atrial fibrillation	1 (0.8)	1.6	1 (1.3)	1.0	26 (8.0)	3.5	20 (6.1)	3.3

[a]An external placebo control arm was included using data from patients who received placebo in the phase 3 APOLLO study. The APOLLO study was completed before the onset of the COVID-19 pandemic. [b]Exposure-adjusted AER or SAER, where applicable, per 100 patient-years calculated as events/patient-year x 100. [c]AEs and serious AEs shown are those that occurred in ≥10% and ≥5% of patients in the combined vutrisiran-treated group in the pooled analysis.

AE = adverse event; AER = adverse event rate; PY = patient-years; SAER = serious adverse event rate.

SUPPLEMENTAL TABLE 4 Incidence of Adverse Events in Patient Subgroups in the Combined Vutrisiran Group

	Combined vutrisiran, n (%)							
	**Age**		**Sex**		**Race**		**ATTR type[a]**	
	<75 years (n = 331)	≥75 years (n = 376)	Male (n = 609)	Female (n = 98)	White (n = 578)	Non-White (n = 129)	ATTRv (n = 214)	ATTRwt (n = 493)
At least 1 AE	302 (91.2)	320 (85.1)	528 (86.7)	94 (95.9)	512 (88.6)	110 (85.3)	206 (96.3)	416 (84.4)
At least 1 SAE	154 (46.5)	177 (47.1)	282 (46.3)	49 (50.0)	274 (47.4)	57 (44.2)	103 (48.1)	228 (46.2)
**AEs occurring in ≥10% of vutrisiran-treated patients in the overall combined group**								
Atrial fibrillation	44 (13.3)	60 (16.0)	91 (14.9)	13 (13.3)	87 (15.1)	17 (13.2)	29 (13.6)	75 (15.2)
Cardiac failure	45 (13.6)	95 (25.3)	126 (20.7)	14 (14.3)	123 (21.3)	17 (13.2)	25 (11.7)	115 (23.3)
COVID-19	84 (25.4)	65 (17.3)	123 (20.2)	26 (26.5)	126 (21.8)	23 (17.8)	51 (23.8)	98 (19.9)
Fall	46 (13.9)	51 (13.6)	78 (12.8)	19 (19.4)	78 (13.5)	19 (14.7)	40 (18.7)	57 (11.6)

[a]Only patients with ATTRv were enrolled in HELIOS-A.

AE = adverse event; SAE = serious adverse event.

SUPPLEMENTAL TABLE 5 **Summary of Cardiac Adverse Events**

	HELIOS-A			HELIOS-B			Combined	
	Vutrisiran n = 160, 539.2 PY		APOLLO Placebo[a] n = 77, 96.1 PY	Vutrisiran n = 547, 979.7 PY		Placebo n = 328, 822.4 PY	Vutrisiran N = 707, 1518.9 PY	
**Parameter**	**n (%)**	**AER[b]**	**AER[b]**	**n (%)**	**AER[b]**	**AER[b]**	**n (%)**	**AER[b]**
**At least 1 adverse event in:**								
Cardiac disorders SOC	58 (36.3)	23.6	46.8	283 (51.7)	65.6	83.5	341 (48.2)	50.7
SAEs in cardiac disorders SOC	25 (15.6)	6.9	15.6	142 (26.0)	24.4	31.5	167 (23.6)	18.2
Cardiac failure SMQ (narrow and broad)	49 (30.6)	15.8	56.2	208 (38.0)	37.6	51.6	257 (36.4)	29.8
Cardiac failure SMQ (narrow)	18 (11.3)	4.6	12.5	167 (30.5)	29.2	42.9	185 (26.2)	20.5
Cardiac arrhythmia HLGT	42 (26.3)	14.8	30.2	167 (30.5)	30.3	33.0	209 (29.6)	24.8

[a]An external placebo control arm was included using data from patients who received placebo in the phase 3 APOLLO study. [b]Exposure-adjusted AER or SAER, where applicable, per 100 patient-years calculated as events/patient-year x 100.

AER = adverse event rate; HLGT = High Level Group Term; PY = patient-years; SAE = serious adverse event; SAER = serious adverse event rate; SMQ = Standardized MedDRA (Medical Dictionary for Regulatory Activities) Queries; SOC = System Organ Class.

SUPPLEMENTAL TABLE 6 **Summary of Thromboembolic Adverse Events**

	HELIOS-A			HELIOS-B			Combined	
	Vutrisiran n = 160, 539.2 PY		APOLLO Placebo[a] n = 77, 96.1 PY	Vutrisiran n = 547, 979.7 PY		Placebo n = 328, 822.4 PY	Vutrisiran N = 707, 1518.9 PY	
**Parameter**	**n (%)**	**AER[b]**	**AER[b]**	**n (%)**	**AER[b]**	**AER[b]**	**n (%)**	**AER[b]**
Deep vein thrombosis	1 (0.6)	0.2	1.0	1 (0.2)	0.3	0.1	2 (0.3)	0.3
Pulmonary embolism	0	0	1.0	4 (0.7)	0.4	0.1	4 (0.6)	0.3

[a]An external placebo control arm was included using data from patients who received placebo in the phase 3 APOLLO study. [b]Exposure-adjusted AER, where applicable, per 100 patient-years calculated as events/patient-year x 100.

AER = adverse event rate; PY = patient-years.

SUPPLEMENTAL TABLE 7 Adverse Events by Organ System During the Initial Randomized Treatment Periods

	HELIOS-A 18-month Treatment Period				HELIOS-B Double-blind Treatment Period			
	Vutrisiran n = 122, 191.3 PY		APOLLO Placebo[a] n = 77, 96.1 PY		Vutrisiran n = 326, 833.9 PY		Placebo n = 328, 822.4 PY	
	n (%)	AER[b]	n (%)	AER[b]	n (%)	AER[b]	n (%)	AER[b]
Cardiac events[c]	37 (30.3)	34.0	28 (36.4)	46.8	227 (69.6)	64.2	242 (73.8)	83.5
Ocular events[d]	35 (28.7)	28.2	20 (20.6)	27.0	47 (14.4)	7.7	66 (20.1)	12.6
Hepatic events[e]	6 (4.9)	4.7	6 (7.8)	7.3	56 (17.2)	10.4	62 (18.9)	10.9
Renal events[f]	5 (4.1)	3.7	9 (11.7)	13.5	55 (16.9)	7.9	57 (17.4)	9.7

[a]An external placebo control arm was included using data from patients who received placebo in the phase 3 APOLLO study. [b]Exposure-adjusted AER per 100 patient-years calculated as events/patient-year x 100. [c]Cardiac disorders SOC. [d]Eye disorders SOC. [e]Mapped to the MedDRA "Drug-related hepatic disorders" comprehensive search SMQ (broad and narrow terms). [f]Mapped to the MedDRA "Acute renal failure" comprehensive search SMQ (broad and narrow terms). If a patient had more than 1 event in a given SOC or SMQ, that patient is counted once for the SOC or SMQ.

AER = adverse event rate; MedDRA = Medical Dictionary for Regulatory Activities; PY = patient-years; SMQ = Standardized MedDRA Queries; SOC = system organ class.
